# Legislative Constraints and Moral Distress: Reframing Occupational Risks Through a Public Health Ethics Lens

**DOI:** 10.1093/phe/phag005

**Published:** 2026-04-27

**Authors:** Elpidio Maria Garzillo, Vincenzo Crispino, Maria Grazia Lourdes Monaco

**Affiliations:** Department of Prevention, Abruzzo Local Health Authority, L’Aquila 67100, Italy; Salerno Local Health Authority, Salerno 84121, Italy; Department of Diagnostic and Public Health, University of Verona, Verona 37134, Italy

## Abstract

This Case for Discussion examines legislative constraints as occupational health hazards that induce moral distress among healthcare professionals (including physicians, nurses, and midwives). Traditionally framed as clinical ethics issues, moral distress emerges as a population-level public health concern when regulatory barriers systematically conflict with clinical judgment. Drawing on empirical evidence from abortion restrictions and European contexts (Italy, France, Spain), the case highlights psychological harms, burnout, and systemic consequences like defensive medicine and workforce emigration. Key normative questions address occupational risk classification, stress assessment tools, prospective legislative impact evaluations, occupational medicine roles, professional associations’ advocacy, and institutional obligations balancing conscientious objection with service provision. The analysis reframes moral distress through a public health ethics lens, advocating expanded risk frameworks, organizational support, and policy interventions to protect healthcare workers while advancing population health goals.

## Background

The moral distress experienced by healthcare professionals has traditionally been understood as a matter of clinical ethics, i.e. the psychological response of an individual professional to situations in which they perceive obstacles to providing ethically appropriate care (Kherbache *et al.*, 2022; Buchbinder *et al.*, 2024). This approach places moral distress within the doctor-patient relationship and emphasizes resolution through clinical ethics consultation, individual coping strategies or institutional ethics committees. However, emerging evidence suggests that this phenomenon warrants re-examination through a public health ethics lens, particularly as an occupational health risk. When legislative constraints systematically prevent healthcare professionals from providing what they consider ethically appropriate care, the resulting psychological harm transcends individual clinical dilemmas and becomes a population-level occupational hazard affecting entire professional groups across jurisdictions (Dean *et al.*, 2019). This reframing shifts the analytical focus from individual moral reasoning to the structural determinants of worker health and safety in the context of public health practice.

## The Case: Legislative Changes as Occupational Stressors

Recent empirical research illustrates this interplay. Sabbath and colleagues documented significant occupational health effects among obstetrician-gynaecologists (OB-GYNs), nurses or midwives following restrictive abortion legislation in several US states (Sabbath *et al.*, 2024). Their findings revealed that such legal prohibitions can cause sleep disturbances, emotional exhaustion, and burnout symptoms in OB-GYNs, directly attributable to legal constraints that conflict with their clinical judgment and professional obligations. Similar patterns emerge in several regulatory areas: medically assisted reproduction, end-of-life care, advance directives, and access to onco-fertility services (Desai *et al.*, 2023). Regulatory changes and conflicting national/regional regulations create bureaucratic burdens and operational difficulties for physicians and other healthcare professionals who must comply with regulations (Lamb *et al.*, 2019; Bosack *et al.*, 2025).

In some European regulatory contexts, particularly in Italy, France and Spain, these stress factors can also affect professionals who play a key role in monitoring workers’ health, such as occupational physicians. As healthcare professionals, they may have to navigate regulatory changes, such as the ‘right to be forgotten’ for cancer survivors, or regional variations in reproductive health legislation, and workers’ rights to health and work in order to fulfill their dual mandate: protecting workers’ well-being and ensuring regulatory compliance (EU-OSHA, 2023). These regulatory constraints can cause moral damage when professional duties conflict with legal limitations, compromising the well-being of doctors, including occupational physicians.

The concept of ‘moral injury’ captures the distinctive nature of these occupational stressors: the psychological distress that arises when legal constraints prevent physicians from providing adequate patient care, compounded by fear of professional liability or institutional repercussions (Reingold *et al.*, 2022). Unlike typical workplace stressors, which can be addressed through ergonomic interventions or structural reorganization, legislative barriers represent systemic risks inherent in the regulatory framework of healthcare practice itself. Beyond individual practitioner wellbeing, these legislative constraints generate system-level consequences: defensive medicine practices that compromise care quality, patient migration to permissive jurisdictions that create access inequities, clinician emigration that depletes workforce capacity, and erosion of institutional trust (McCarthy *et al.*, 2023). From an occupational health perspective within public health ethics, these patterns suggest legislative changes function as upstream determinants of workers’ health, with downstream implications for population health.

## Normative Questions for Public Health Ethics

All these observations raise several issues that deserve to be analysed within the ethical frameworks of public health, as explained below.

- Within the framework of the reference classification of occupational risks, how should legislative constraints is categorized in occupational risk assessment systems? Should they be classified as psychosocial hazards comparable to organizational stressors, or do they constitute a distinct category of regulatory-induced occupational risks requiring specialized evaluation methods?
- If legal and regulatory constraints are taken into account in the assessment of work-related stress, according to European Framework Directive 89/391/EEC, should they be explicitly included as potential stress factors? What validated tools could detect the moral distress induced by legislation alongside traditional workplace stress factors?
- Should legislative processes affecting healthcare practice include prospective occupational health impact assessments? What mechanisms could ensure policymakers consider potential harms to healthcare worker wellbeing when designing regulatory constraints?
- How can occupational medicine help healthcare workers deal with these complex situations in which regulatory frameworks themselves could generate occupational risks?
- How should professional medical associations protect the clinical autonomy of doctors, promoting regulatory changes where necessary, and ensure that the health priorities of the population expressed in legislation are balanced with the occupational health rights of the professionals who provide care?
- Ultimately, what obligations do healthcare institutions have toward workers who face legislative constraints? Drawing on the Canadian concept of ‘conscientious provision’ developed in the context of medical assistance in dying, should institutions be required to create environments that enable doctors to provide standard care within legal limits, including transfers, referrals and protection policies? How can public health ethical frameworks address the tension between physicians’ individual rights, such as to conscientious objection, and institutional obligations to ensure the workforce's ability to provide legally permitted services? What models exist to protect both physicians’ consciences and patients’ access?

## Implications for Public Health Ethics Research and Practice

This Case for Discussion invites the public health ethics community to consider whether current regulatory frameworks adequately address the occupational health dimensions of legislative constraints on healthcare practice, and what theoretical and practical developments might better protect healthcare workers while promoting population health goals.

In more detail, this case demonstrates that redefining moral distress as an occupational health risk does not detract from the harms suffered by patients subject to restrictive legislation (e.g. reproductive health harms resulting from abortion bans), but rather adds an additional analytical layer that foregrounds the protection of healthcare workers as a public health priority. As occupational health is a fundamental area of public health research and practice, we propose that risk assessments broaden their scope to include legislative factors that may be considered workplace stressors, while healthcare organizations should simultaneously provide comprehensive support, including legal advice, psychological services, professional development, and workplace wellbeing programs. Professional associations, in turn, have a responsibility to defend clinical autonomy while remaining within legal boundaries.

Occupational medicines, particularly in European regulatory contexts emphasizing Worker Health Promotion, are uniquely positioned to contribute to this discourse. Their statutory role—monitoring workers’ physical and psychological health through individual assessments, validated screening tools, and early intervention—positions them as critical observers of legislative impacts on workforce wellbeing. They remain uniquely positioned to advocate for healthcare worker protection, ensuring legislative frameworks support rather than undermine occupational health and safety. Their expertise in risk assessment, exposure surveillance, and preventive interventions offers evidence-based approaches to mitigating legislative-induced occupational hazards.
